# The new frontline: exploring the links between moral distress, moral resilience and mental health in healthcare workers during the COVID-19 pandemic

**DOI:** 10.1186/s12888-021-03637-w

**Published:** 2022-01-06

**Authors:** Edward G. Spilg, Cynda Hylton Rushton, Jennifer L. Phillips, Tetyana Kendzerska, Mysa Saad, Wendy Gifford, Mamta Gautam, Rajiv Bhatla, Jodi D. Edwards, Lena Quilty, Chloe Leveille, Rebecca Robillard

**Affiliations:** 1 Department of Medicine, University of Ottawa and Clinical Epidemiology Program, Ottawa Hospital Research Institute, 1053 Carling Avenue, Ottawa, Ontario K1Y 4E9 Canada; 2 Berman Institute of Bioethics & School of Nursing, Johns Hopkins University, 1809 Ashland Avenue, Baltimore, MD 21205 USA; 3 The University of Ottawa Institute of Mental Health Research at The Royal, 1145 Carling Avenue, Ottawa, Ontario K1Z 7K4 Canada; 4 Faculty of Medicine, University of Ottawa, 1145 Carling Avenue, Ottawa, Ontario K1Z 7K4 Canada; 5 Faculty of Health Sciences, School of Nursing, University of Ottawa, 451 Smyth Road, Ottawa, Ontario, K1H 8M5 Canada; 6 Royal Ottawa Mental Health Centre, 1145 Carling Avenue, Ottawa, Ontario K1Z 7K4 Canada; 7 Brain and Heart Nexus Research Program, University of Ottawa Heart Institute, 40 Ruskin Street, Ottawa, Ontario K1Y 4W7 Canada; 8 School of Epidemiology and Public Health, University of Ottawa, 600 Peter Morand Crescent, Room 101, Ottawa, Ontario K1G 5Z3 Canada; 9 Centre for Addiction and Mental Health, 1025 Queen Street, Toronto, Ontario, M6J 1H1 Canada; 10 School of Psychology, University of Ottawa, 136 Jean-Jacques Lussier, Ottawa, Ontario K1N 6N5 Canada

**Keywords:** Healthcare workers, Moral distress, Moral resilience, Mental health, Stress, Anxiety, Depression, Pandemic, Global crisis

## Abstract

**Background:**

Global health crises, such as the COVID-19 pandemic, confront healthcare workers (HCW) with increased exposure to potentially morally distressing events. The pandemic has provided an opportunity to explore the links between moral distress, moral resilience, and emergence of mental health symptoms in HCWs.

**Methods:**

A total of 962 Canadian healthcare workers (88.4% female, 44.6 + 12.8 years old) completed an online survey during the first COVID-19 wave in Canada (between April 3rd and September 3rd, 2020). Respondents completed a series of validated scales assessing moral distress, perceived stress, anxiety, and depression symptoms, and moral resilience. Respondents were grouped based on exposure to patients who tested positive for COVID-19. In addition to descriptive statistics and analyses of covariance, multiple linear regression was used to evaluate if moral resilience moderates the association between exposure to morally distressing events and moral distress. Factors associated with moral resilience were also assessed.

**Findings:**

Respondents working with patients with COVID-19 showed significantly more severe moral distress, anxiety, and depression symptoms (F > 5.5, *p* < .020), and a higher proportion screened positive for mental disorders (Chi-squared > 9.1, *p* = .002), compared to healthcare workers who were not. Moral resilience moderated the relationship between exposure to potentially morally distressing events and moral distress (*p* < .001); compared to those with higher moral resilience, the subgroup with the lowest moral resilience had a steeper cross-sectional worsening in moral distress as the frequency of potentially morally distressing events increased. Moral resilience also correlated with lower stress, anxiety, and depression symptoms (*r* > .27, *p* < .001). Factors independently associated with stronger moral resilience included: being male, older age, no mental disorder diagnosis, sleeping more, and higher support from employers and colleagues (B [0.02, |-0.26|].

**Interpretation:**

Elevated moral distress and mental health symptoms in healthcare workers facing a global crisis such as the COVID-19 pandemic call for the development of interventions promoting moral resilience as a protective measure against moral adversities.

**Supplementary Information:**

The online version contains supplementary material available at 10.1186/s12888-021-03637-w.

## Background

Healthcare workers (HCWs) are commonly exposed to elevated stress and exhaustion, and a myriad of ethical conflicts and dilemmas that can degrade their health and wellbeing. Moral distress arises when HCWs face moral adversity, must make a moral judgment about the most ethically justified response, and act on it in a situation where the consequences of action (or inaction) imperil their moral integrity [1]. While the world’s healthcare systems are struggling to cope with the COVID-19 pandemic, systemic failure to safeguard HCWs from developing moral distress, or effectively helping manage it, risks creating a workforce gap in healthcare delivery. HCW’s absenteeism or quitting their professions completely, which can take years to replace, have immediate and long-term effects on the standards of care. Mental health symptoms (stress, anxiety, burnout, depression) have increased in HCWs facing global crises like the COVID-19 pandemic [2–6], presenting a unique lens through which to assess the potential relationship between external global stressors, moral distress and mental health.

The COVID-19 pandemic has had major collateral effects on the global healthcare system creating sustained and unrelenting pressure to re-allocate scarce healthcare resources including HCWs. During the pandemic, many HCWs have encountered potentially morally distressing events (PMDEs) over and above the stressors faced during their typical practice, such as risk of COVID-19 transmission to family members, caring for patients without family members present, triaging patients in the context of limited resources where the lack of treatment may result in death, and following directions that go against their professional standards or core values [7, 8]. Accordingly, moral distress in HCWs during the COVID-19 pandemic has been shown to be high [9, 10]. Amongst its other adverse effects on HCWs, this pandemic can thus be conceptualized as a global trigger for surging PMDEs, a phenomenon that may be commensurate to the degree of exposure to patients with COVID-19.

**Fig. 1:**
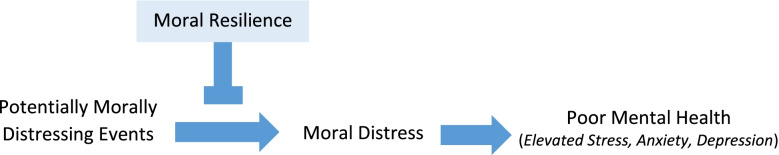
Theoretical model

Concerns have been raised about the risks associated with moral distress and moral injury faced by HCWs during the COVID-19 pandemic [11, 12]. Moral distress was found to relate to the volume of care of patients infected with COVID-19, access to personal protective equipment, and communication from leaders [8]. Working in a stressful, less supportive environment during the COVID-19 pandemic has been associated with increased moral injury [12]. While a link has been proposed between moral distress/injury and adverse mental health outcomes [13, 14], there is little empirical data and a lack of knowledge about potential factors that may help prevent and better manage moral distress in HCWs. Moral resilience, which refers to the capacity to sustain or restore one’s integrity in response to moral adversity [15], has been proposed as a pathway to mitigate the detrimental effects of moral adversity [16]. Sharing similar features with general resilience, moral resilience specifically incorporates individual factors that can help HCWs practice in a manner that reflects their intentions, character, and integrity [15, 17] while confronting an ethically adverse situation without lasting detrimental effects of moral distress. Given the recent emergence of the concept of moral resilience, we conceptualized an innovative model postulating that: i) moral resilience moderates the degree of moral distress caused by PMDEs and ii) lower moral distress arising from PMDEs would be associated with lower downstream mental health burden (Fig. 1).

To test this model, we conducted a cross-sectional study to measure PMDEs, levels of moral distress, mental health symptoms, and moral resilience in HCWs during the COVID-19 crisis. Our specific research objectives were to: 1) Characterize the degree of moral distress and other mental health outcomes in healthcare workers during the COVID-19 pandemic based on exposure to patients with COVID-19; 2) Determine whether moral resilience moderates the association between exposure to morally distressing situations and ensuing moral distress; 3) Evaluate how mental health relates to moral distress and resilience; 4) Identify factors associated with stronger moral resilience.

## Methods

Data for this study was collected as part of a larger online survey (ClinicalTrials.gov: NCT04369690) aiming to assess the occupational, financial and psychosocial impacts of the COVID-19 pandemic [18]. The survey was programmed and managed using an online platform (Qualtrics, Provo, UT) and distributed between April 3rd and September 3rd, 2020 (i.e. during the first wave of the COVID-19 pandemic in Canada) via websites, social media, and multiple organizations and hospitals across Canada. The survey was available in both English and French and was developed and conducted following guidelines from the Checklist for Reporting Results of Internet E-Surveys (CHERRIES) [19]. The exact date on which each individual survey was done was documented based on an automated timestamp from the survey platform. This timestamp was used to calculate the time elapsed since the pandemic declaration by the World Health Organization on March 11, 2020.

### Participants

Adults who self-identified as healthcare professionals or healthcare administrators were recruited from social media, staff email lists and newsletters from several hospitals, and health-related organizations (please see full list in acknowledgements). The only inclusion criteria were to self-identify as an HCW, to be located within Canada and to have sufficient data for the main study outcomes (less than 25% of items missing). There were no exclusion criteria. These HCWs were not restricted to staff working with patients diagnosed with COVID-19.

### Questionnaires

Full details on the questions included in this survey have previously been reported [18]. In brief, demographic questions notably covered profession types (i.e. participants had to select from a list of common professions (please see details in Table 1) and exposure to patients with COVID-19 (i.e. “Does your work currently involve contact (in person) with people who tested positive for COVID-19?”). The presence of self-reported diagnoses of a mental disorder (i.e. “Have you ever had a formal diagnosis of any mental disorder (e.g. Anxiety disorder, depression)?”) was also documented through this survey as this is bound to influence mental health symptoms. Furthermore, the survey included custom-made questions about perceived social support (i.e. “How much do you agree with the following statements - Since the beginning of the outbreak, I have experienced significant levels of support from: i) my family, ii) my employer, colleagues”; scale from 1 - strongly disagree to 11 - strongly agree) and sleep (i.e. item derived from the Pittsburgh Sleep Quality Index [20] “In the past 7 days, how many hours of actual sleep did you get at night (This may be different than the number of hours you spent in bed.)”).

**Table 1:** Sample characteristics

	*n*	Mean/ Fr	SD/ %
Time elapsed since the pandemic began (days)	962	65.6	29.9
General Demographics			
Age (years)	962	44.6	12.8
Biological sex (Females)	962	850	88.4%
Ethnicity (Caucasian)	575	492	85.6%
Usual number of work hours per week	881	36.5	12.2
Contact with COVID-19 Patients (vs none)	915	291	31.8%
Total Family Income (> 100 K)	580	386	66.6%
Has underage children (vs none)	939	282	30.0%
Current province/territory	866		
Ontario		641	74.0%
Quebec		196	22.6%
Northwest Territories		10	1.2%
Manitoba		7	0.8%
British Columbia		6	0.7%
Alberta		2	0.2%
Saskatchewan		2	0.2%
Nova Scotia		1	0.1%
Nunavut		1	0.1%
Psychological Factors			
History of mental disorder diagnosis (vs none)	930	305	32.8%
Current mental disorder diagnosis (vs none)	915	195	21.3%
Previously exposed to major stressor (vs not)^a^	934	424	45.4%
Work-related factors			
Contact with COVID-19 patients (vs not)	962	630	65.5%
Reallocated a different unit/discipline since pandemic	962	192	20.0%
Shift work (vs none)	909	261	28.7%
HCW type	962		
Nurse		270	28.1%
Allied Mental Health Specialist		214	22.2%
Other Allied Health^b^		189	19.6%
Physician		163	16.9%
Administrator		126	13.1%
Discipline	355		
Psychiatry		40	11.3%
Cardiology/Thoracic Surgery^c^		25	7.0%
Emergency Medicine		25	7.0%
Geriatrics		24	6.8%
Other^d^		21	6.0%
Critical Care Medicine		19	5.4%
Pediatrics		19	5.4%
General Internal Medicine		16	4.5%
Diagnostics^e^		14	4.1%
Family Medicine		14	3.9%
Obstetrics/ Gynecology		12	3.4%
Public Health and Preventive Medicine		12	3.4%
Oncology		10	2.8%
Nephrology		8	2.3%
Respiratory Medicine/ Respirology		5	1.4%
Neurosurgery		4	1.1%
Other^f^		87	24.5%

Means, standard deviation (SD), frequencies (Fr), and percentages (%) of main demographic variables for the global sample.

^a^Refers to a natural disaster, fire/ explosion, transport accident, physical or sexual assault, combat/ exposure to a war zone, life-threatening illness, or injury.

^b^Other Allied Health: e.g. pharmacist, respiratory therapist, medical radiation technologist, physiotherapist.

^c^Cardiology/Thoracic Surgery: Cardiology (*n*=20, 5.6%), Cardiovascular/Thoracic Surgery (*n*=5, 1.4%).

^d^Other: Anesthesiology (*n*=3, 0.8%), Gastroenterology (*n*=2, 0.6%), Ophthalmology (*n*=2, 0.6%), Orthopedic Surgery (*n*=3, 0.8%), Otolaryngology (*n*=1, 0.3%), Physical Medicine and Rehabilitation (*n*=2, 0.6%), Plastic Surgery (*n*=2, 0.6%), Radiation Oncology (*n*=2, 0.6%), Rheumatology (*n*=3, 0.8%), Urology (*n*=1, 0.3%)

^e^Diagnostics: Anatomical Pathology (*n*= 2, 0.6%), Diagnostic Radiology (*n*=1, 0.3%), Endocrinology/ Metabolism (*n*=2, 0.6%), General/ Clinical Pathology (*n*=1, 0.3%), Hematology (*n*=4, 1.1%), Medical Microbiology and Infectious Diseases (*n*=2, 0.6%), Nuclear Medicine (*n*=2, 0.6%).

^f^Other: e.g. long-term care, palliative care, general surgery, float nurse.

The survey included the Measure of Moral Distress for Health Care Professionals (MMD-HP) [21], a revision of the widely used Moral Distress Scale-Revised (MDS-R) [22] adapted for HCWs. The MMD-HP counts 27 items measuring current levels of moral distress as a function of how often a situation occurs and how distressing it is. Respondents rate each item on two Likert scales to indicate: how often a situation occurs during their practice (frequency: 0 = never, 4 = very frequent) and how distressing it is when it occurs (distressing: 0 = none, 4 = very distressing). Standard scoring procedures for this scale involve multiplying the frequency score (f) by the distress score (d) to generate a composite score for each item (“f x d”, range 0–16). These composite scores are then summed to create the overall MMD-HP score (ranging from 0 to 432), with higher scores indexing higher moral distress. In addition to the overall MMD-HP score, the MMD-HP frequency score was used to characterize the degree of exposure to PMDEs since it effectively quantifies the frequency of exposure to various situations which are potentially morally distressing. The MMD-HP was found to have good reliability for different subtypes of HCWs (α = 0.93) and to perform similarly to the MDS-R [21].

The recently developed Rushton Moral Resilience Scale (RMRS) [23] contains 17 items assessing the core components of moral resilience in HCWs: (response to moral adversity, personal integrity, relational integrity, and moral efficacy (see example items in supplementary materials). Participants are asked to rate how much they agree with each item on a Likert scale (from 1 = disagree to 4 = agree). A total score is calculated by finding the mean of all 17 items, with higher total scores indicating greater moral resilience. The RMRS has good overall reliability (α = 0.84) and demonstrated convergent validity with the Connor Davidson Resilience Scale-10 and criterion validity with the Maslach Burnout Inventory–Human Services Survey [23, 24].

The survey also included the Generalized Anxiety Disorder Scale (GAD-7) [25], the Quick Inventory of Depressive Symptomatology-Self Report (QIDS-SR16) [26, 27], and the Perceived Stress Scale (PSS) [28]. Total scores were computed for PSS, GAD-7, and QIDS-SR16 and used as continuous variables for variance and correlation analyses. These scores were also used to classify participants according to positive screens based on established criteria (i.e. PSS > 13, GAD-7 > 10 and QIDS-SR16 > 13) for analyses based on contingency tables.

### Statistical analyses

To address the first aim, chi-squared tests were used to compare the proportion of individuals with self-reported current diagnoses of mental disorders in the subgroups with and without exposure to patients with COVID-19 (coded as a binary variable). The severity of moral distress, stress, anxiety, and depression symptoms (total scores on the MMD-HP, PSS, GAD-7 and QIDS-SR16 respectively) was compared across these two groups with ANCOVAs controlling for age and self-reported current mental disorder diagnosis. Chi-squared tests were used to compare the proportion of individuals within each MMD-HP tertile and those screening positive for mental health symptoms (i.e. PSS > 13, GAD-7 > 10 and QIDS-SR16 > 13). For the second aim, a multiple linear regression using the enter method [29] was designed to test if moral resilience moderates the association between the frequency of exposure to morally distressing situation and ensuing moral distress. This model included MMD-HP total score as the dependent variable and the following explanatory variables: the frequency of exposure to PMDEs (frequency score on the MMD-HP), moral resilience (RMRS total score), a statistical interaction term between the frequency of exposure to PMDEs and moral resilience, and covariates which could influence these factors [i.e. the time elapsed since the pandemic declaration, sex, age, the presence of a current diagnosed mental disorder (coded as a binary variable), and profession type (physician, nurse, allied mental health specialist, other allied health or healthcare administrator; coded as dummy variables)]. For the third aim, partial correlations were conducted to determine if moral distress (MMD-HP total score) and moral resilience (RMRS total score) are associated with the severity of perceived stress (PSS), anxiety (GAD-7), and depression (QIDS-SR16) symptoms while controlling for the same covariates listed above. To address the fourth aim, a multiple linear regression model was used to assess how moral resilience is associated with: age, sex, the presence of a current diagnosed mental disorder, HCWs subtypes, sleep duration over the past 7 days, and the degree of social support received from one’s family and their employer/colleagues (treated as continuous variables). Analyses were conducted using the Statistical Package for Social Sciences (IBM SPSS Statistics for Windows, V.23.0. Armonk, USA).

## Results

### Sample characteristics

Sample descriptives are presented in Table 1. Overall, 962 HCWs took part in this study. The sample was 88.4% female, 85.6% Caucasian, and ranged from 18 to 80 years of age (mean [*M*] = 44.6, standard deviation [*SD*] = 12.8 years). Most respondents were located in Ontario (74.0%, *n* = 641) or Quebec (22.6%, *n* = 196), and included a higher proportion of nurses (28.1%, *n* = 270), followed by allied mental health specialists (22.2%, *n* = 214), allied health specialists (19.6%, *n* = 189), physicians (16.9%, *n* = 163), and administrators (13.1%, *n* = 126). A higher proportion of participants were working in psychiatry (11.3%, *n* = 40), emergency medicine (7.0%, *n* = 25), and cardiology/thoracic surgery (7.0%, *n* = 25) compared to other disciplines. Overall, 26.8% of respondents (*n* = 255) had been tested for COVID-19 prior to survey completion, and out of those, 5.5% indicated that they had tested positive (*n* = 14). Within the global sample, 31.8% (*n* = 291) reported that their work involved contact with patients diagnosed with COVID-19. These respondents had similar length of time elapsed since the start of the pandemic to survey completion and similar distributions in terms of sex, race, income level, and past trauma exposure compared to those who had not been in contact with patients with COVID-19 (Table S1, all *p* > .050). Conversely, those who were in contact with COVID-19 positive patients were significantly younger (39.6 + 11.3 vs 46.5 + 12.7 years old; F (1, 913) = 62.9, *p* < .001, np2 [partial eta squared, an estimate of effect size] = .064).

### Moral distress and mental health classified by exposure to patients with COVID-19

A higher proportion of HCWs in contact with patients with COVID-19 reported having a current diagnosis of a mental disorder (27.4%, *n* = 76/277) compared to those who were not in contact with COVID-19 positive patients (18.5%, *n* = 110/593; Chi-squared = 8.9, *p* = .003).

**Fig. 2:**
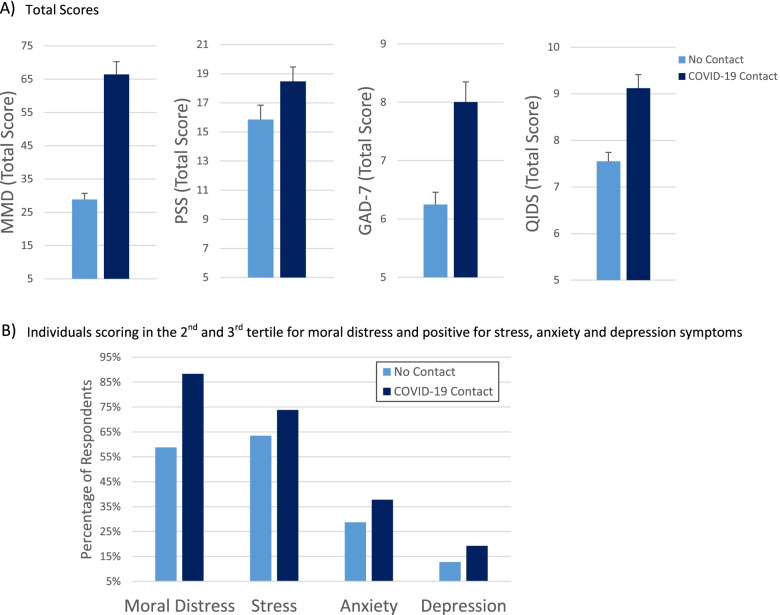
Moral distress and mental health based on exposure to patiets with COVID-19

After controlling for age and self-reported current mental disorder diagnoses, the severity of moral distress (F (1, 866) = 69.9, *p* < .001, np2 = .075), anxiety (F (1, 833) = 5.5, *p* = .020, np2 = .007), and depression (F (1, 805) = 5.5, *p* = .020, np2 = .007) were significantly higher in HCWs exposed to COVID-19 positive patients compared to those who were not (Fig. 2A). No significant effect was found for stress (PSS, F (1, 767) = 3.6, *p* = .058, np2 = .005).

The proportion of individuals falling within the second or third tertile of moral distress (i.e. higher levels of moral distress) and the proportion of those screening positive for stress, anxiety and depression were higher in respondents who were in contact with patients with COVID-19 compared to those who were not (Chi-squared > 9.1, *p* = .002, Fig. 2B).

### Moral resilience moderates the relationship between exposure to potentially morally distressing events and moral distress

**Fig. 3:**
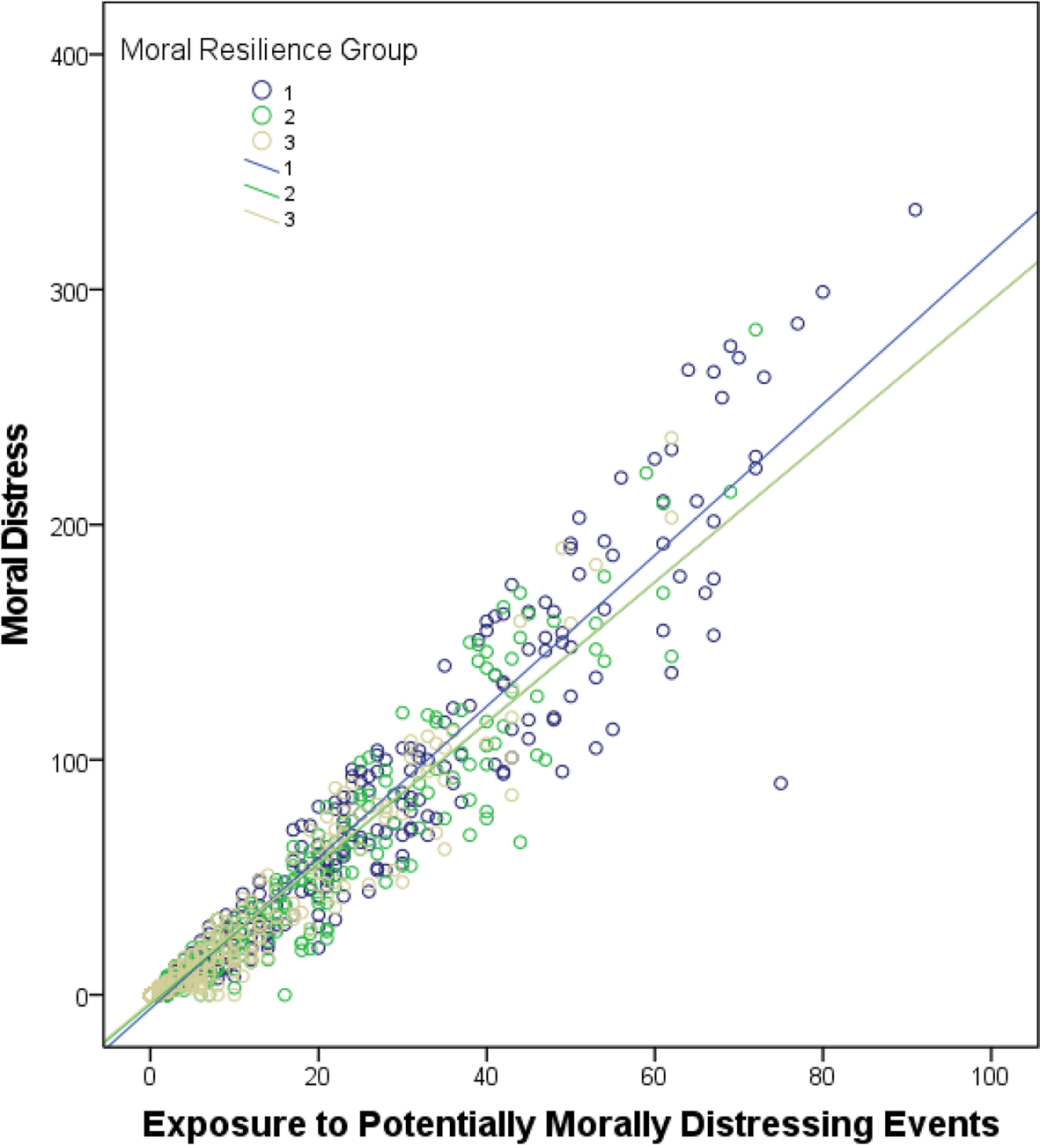
Relationship between moral resilience, exposure to morally distressing events and moral distress

In the multiple linear regression model aiming to assess moderation, higher exposure to PMDEs (B = 2.98, 95% CI [2.90, 3.05]) and lower moral resilience (B = 3.12, 95% CI [0.04, 6.20]) were associated with more severe moral distress (Table 2). The R2 of the model integrating the interaction term (exposure by moral resilience) was significantly higher than the main effects model (R2 = .93 vs .92; F Change (1, 898) = 52.6, *p* < .001). The interaction term between PMDEs exposure and moral resilience was significantly associated with moral distress. As can be seen on Fig. 3, moral resilience moderated the positive association between exposure to PMDEs and moral distress. Specifically, compared to subgroups with higher moral resilience (second and third tertiles), the subgroup with the lowest moral resilience (first tertile) had a steeper cross-sectional worsening in moral distress as the frequency of PMDEs increased (i.e. individuals with more frequent PMDEs were more prone to have higher moral distress if their level of moral resilience was low). The multiple regression model also revealed that older age (B = 1.22, 95% CI [0.41, 2.02]) was associated with higher levels of moral distress. There was no significant independent association between moral distress and the time elapsed since the pandemic declaration.

**Table 2:** Moderation model

			95% CI		
	B	SE	LL	UL	p
Time Elapsed since the pandemic declaration (per 7 days)	−0.22	0.12	−0.45	0.02	.072
Demographics					
Male Sex (vs Female)	−0.50	1.61	−3.65	2.65	.756
Age (per 10 years increase)	1.22	0.41	0.41	2.02	.003
Current mental disorder (vs none)	1.44	1.26	−1.03	3.91	.254
Profession (vs Health Administrators)					
Physician	−1.68	1.91	−5.42	2.06	.378
Nurse	0.19	1.74	−3.23	3.61	.913
Other Allied Health	−2.33	1.78	−5.84	1.17	.191
Allied Mental Health Specialist	−0.72	1.76	−4.18	2.73	.681
Moral Factors					
Exposure to PMDEs (Scale 0 to 96)	2.98	0.04	2.90	3.05	<.001
Moral Resilience (Scale 1 to 68)	3.12	1.57	0.04	6.20	.047
Interaction Resilience/Exposure	−0.45	0.06	−0.57	−0.32	<.001

Coefficients from the multiple linear regression assessing moderation of the association between exposure to potentially morally distressing events (PMDEs; Frequency score on the MMD-HP) and moral distress (total score on the Moral Distress for Healthcare Professionals (MMD-HP)) by moral resilience (total score on the Rushton Moral Resilience Scale (RMRS)). B: Unstandardized coefficients (calculated per one unit for continuous variables, except for the time elapsed since the pandemic declaration (which was calculated for each 7 days) and age (which was calculated per 10 years). Units (for continuous variables) and reference groups (for categorical variables) are presented in parenthesis in the first column. *SE* standard error of B, *CI* confidence interval, *LL* lower limit, *UL* upper limit.

### Associations between moral distress/resilience and mental health

After controlling for relevant covariates, higher levels of moral distress were modestly but significantly correlated with more severe stress (PSS, *n* = 763, *r* = .29, *p* < .001), anxiety (GAD7, *n* = 829, *r* = .28, *p* < .001), and depression (QIDS-SR16, *n* = 801, *r* = .27, *p* < .001) symptoms. These correlations persisted after controlling for the degree of moral resilience, although correlation coefficients dropped to lower values (PSS, *n* = 758, *r* = .18, *p* < .001; GAD7, *n* = 823, *r* = .19, *p* < .001; QIDS-SR16, *n* = 796, *r* = .17, *p* < .001).

Higher moral resilience correlated with better mental health outcomes as reflected by lower stress (PSS, *n* = 763, *r* = .29, *p* < .001), anxiety (GAD7, *n* = 829, *r* = .28, *p* < .001), and depression (QIDS-SR16, *n* = 801, *r* = .27, *p* < .001) symptoms (Fig. S1).

### Factors associated with moral resilience

Stronger moral resilience was significantly associated with: being a male (B = 0.12, 95% CI [0.03, 0.21]), older age (B = 0.08, 95% CI [0.05, 0.10]), not having a self-reported current diagnosis of a mental disorder (B = − 0.19, 95% CI [− 0.26, − 0.12]), sleeping more (B = 0.02, 95% CI [0.00, 0.04]), and higher level of support from one’s employer and colleagues (B = 0.12, 95% CI [0.06, 0.17]; Table 3). Conversely, there was no significant independent association between moral resilience and family support, HCW subtype, or the time elapsed since the pandemic declaration.

**Table 3:** Factors associated with moral resilience

			95% CI		
	B	SE	LL	UL	p
Time Elapsed since the pandemic declaration (per 7 days)	<0.01	<0.01	<0.01	0.01	.592
Demographics					
Male Sex (vs Female)	0.12	0.05	0.03	0.21	.008
Age (per 10 years increase)	0.08	0.01	0.05	0.10	<.001
Current mental disorder (vs none)	−0.19	0.04	− 0.26	− 0.12	<.001
Profession (vs Health Administrators)					
Physician	−0.02	0.05	−0.13	0.08	.654
Nurse	−0.07	0.05	−0.17	0.02	.128
Other Allied Health	−0.05	0.05	−0.15	0.05	.301
Allied Mental Health Specialist	0.07	0.05	−0.03	0.17	.166
Social support since the beginning of the outbreak					
Family (Scale 1–11, per 5 points increase)	0.05	0.03	<0.01	0.11	.057
Employer and Colleagues (Scale 1–11, per 5 points increase)	0.12	0.03	0.06	0.17	<.001
Usual sleep duration (per 1 h increase)	0.02	0.01	<0.01	0.04	.048

Coefficients from the multiple linear regression for moral resilience (i.e. total score on the … (RMRS)). B: Unstandardized coefficients (calculated per one unit for continuous variables, except for the time elapsed since the pandemic declaration (which was calculated for each 7 days), age (which was calculated per 10 years), and social support ratings (calculated per 5-points increase). Units (for continuous variables) and reference groups (for categorical variables) are presented in parenthesis in the first column. *SE* standard error of B, *CI* confidence interval, *LL* lower limit, *UL* upper limit

## Discussion

Our findings provide empirical evidence about the importance of moral resilience for the mental health of HCWs facing a global crisis. In our Canadian sample, there were significantly more frequent PMDEs, higher levels of moral distress, more severe stress, anxiety and depression symptoms, and higher occurrences of diagnosed mental disorders in HCWs exposed to patients with COVID-19 compared to those who were not. Consistent with other studies [11, 30, 31], we observed that the frequency and intensity of situations that imperil integrity produce higher levels of moral distress. Most importantly, we observed that moral resilience attenuated the association between the frequency of exposure to PMDEs and ensuing levels of moral distress. HCWs have limited control over their exposure to sources of moral adversity, particularly during a pandemic. The frequency of morally distressing events is likely influenced by the healthcare organization and its ethical climate [32]. Until there are major cultural and organizational shifts in healthcare, HCWs will continue to be exposed to situations creating moral conflict and even more so in periods of additional challenges such as pandemics. What has not been reported previously is the potential protective or buffering effect of moral resilience on the psychological impacts of PMDEs.

This is the first study to use a validated moral resilience scale to assess how it relates to moral distress and mental health symptoms. Our results validated our proposed model, revealing that moral resilience significantly moderates the association between exposure to PMDEs and ensuing moral distress, and that lower moral distress is in turn linked to better mental health. Compared to subgroups with higher moral resilience, the subgroup with the lowest moral resilience had a steeper cross-sectional worsening in moral distress as the frequency of exposure to PMDEs increased. In other terms, individuals who had been more frequently exposed to PMDEs were more prone to commensurate levels of moral distress if their moral resilience was low. Furthermore, higher moral resilience was correlated with lower stress, anxiety and depression symptoms. Altogether, this suggests that moral resilience may be especially important to limit the accumulation of moral distress in the context of a major stressor (such as a pandemic) with beneficial effects on wider mental health outcomes. This suggests that specific strategies to address the moral dimension of clinical care are needed since PMDEs are triggers for moral distress and poor mental health.

Our findings suggest that females, younger people and those with existing mental disorders may be more prone to lower moral resilience. Younger nurses may be particularly at risk for the detrimental effects of moral adversity and moral distress [33]. As they are growing their competence in clinical care, they are confronted with complex ethical challenges that many are not fully prepared to address. Nursing, a predominately female profession, may have additional risks for developing detrimental responses to PMDEs that may be intensified especially in younger individuals. Across the spectrum of healthcare professions, there is a need for pre-licensure curricula to intensify content related to ethical competence and moral resilience, and for programs to extend these skills into clinical practice. Further research is needed to understand how age and perhaps years of experience may impact the development of moral resilience.

Moral resilience is premised on the belief that certain types of moral adversity in high stakes contexts (e.g. pandemic) are unavoidable and in some cases not modifiable. Enhancing moral resilience may help individuals restore their moral agency and autonomy when facing PMDEs. Self-regulatory skills such as mindfulness, tools to amplify certain dimensions of moral resilience like moral efficacy, and practices that foster self-stewardship and buoyancy can restore stability and agency so that HCWs can experience less distress [34, 35]. The goal of reducing distress is not aimed at creating complacency or tolerance of unethical practices or moral adversity, but rather to acknowledge and confront the sources of distress and to provide resources to reduce their short- and long-term detrimental effects [15]. Doing so may reduce the build-up of harmful moral residue and consequent risk of burnout, depression and secondary post-traumatic stress disorder (PTSD) [36].

We observed that higher moral resilience is associated with greater support from employers/co-workers. Hence, bolstering relational integrity, being true to one’s values while respecting the values of others, and embracing shared moral endeavor and interconnection, may be ways to amplify individual moral resilience within the context of the clinical team or organization [15]. Individual integrity is intertwined with the integrity of those served and of those with whom HCWs collaborate to deliver care including colleagues, organizations and the broader society. This finding is counter to claims that focusing on moral resilience puts undue burden on individuals and diverts attention from systemic contributions to the sources of moral adversity in healthcare [37]. Instead of advocating for an “either/or” approach, interventions to mitigate the impact of morally distressing events may benefit from a focus on both on reducing the frequency and intensity of exposure at the systemic level and cultivating the capacities of moral resilience as a protective factor while systemic reforms are implemented [15]. In addition to enhancing support from the employer and colleagues, strengthening institutional structures to identify and respond to ethical concerns, creating spaces for dialogue and discussion [38], structured debriefings about troublesome cases, and peer to peer support led by facilitators trained in the nuances of ethical practice may amplify the foundation of moral resilience. Interventions such as the Mindful, Ethical Practice and Resilience Academy (MEPRA) [35] or other programs aimed at proactively developing and strengthening the capacities associated with moral resilience may provide a needed resource for HCWs.

A holistic approach to HCWs wellbeing may have collateral impact on the ability of HCWs to meet the ethical challenges in their work. Notably, we identified that longer sleep time was associated with stronger moral resilience, a finding aligned with the fact that sleep actively contributes to cognitive and emotional functioning [39]. When sleep deprived, HCWs’ ability to accurately perceive the moral contours of PMDEs and to exercise their moral agency may be dampened. Longer shifts and increased workplace stress due to the pandemic can lead to sleep deprivation and emotional exhaustion, which are both major components of adverse mental health and burnout [40]. Specific strategies to better cope with shiftwork and partial sleep deprivation due to extended work hours when unavoidable, may be relevant means to enhance moral resilience and mental health in HCWs.

Importantly, our findings indicate that longer time elapsed since the onset of the pandemic was not significantly associated with a reduction in the degree of moral distress, suggesting that the situation was not improving as time passed during the first wave of the pandemic. This emphasizes that there may be a need for healthcare organizations and HCWs to incorporate long-term strategies for care during the pandemic and sustain investments in multipronged resources that are needed to support HCWs' well-being and integrity. Notably, creating confidential and proactive mental health screening and removing the stigma associated with mental health treatments may provide long-term benefits [41, 42].

This study has several limitations. Unequal sex and HCW subtype distributions, large proportion of respondents located in a few provinces (Ontario and Quebec), and potential sampling biases limit generalizability. All study data, including HCW status and mental health diagnoses, were based on self-report. Results are also likely to be affected by unmeasured bias inherent to observational studies and the study design does not allow inferences of causality. The use of the MMD-HP to assess both PMDEs and moral distress may seem circular. However, the main objective of this study was not to assess the relationship between these two constructs, which are expected to be highly correlated, but to see if this expected relationship is mediated by a third independent factor: moral resilience. Nevertheless, future studies should replicate these findings with distinct scales. Whilst the majority of evidence about moral injury contributing to PTSD [43] and burnout has been described in military personnel [44–47], this phenomenon needs to be further studied in HCWs.

## Conclusion

Our results confirm that moral resilience, a measurable and potentially modifiable factor, moderates the association between exposure to PMDEs and the ensuing level of moral distress. Greater levels of moral resilience results in lower moral distress and this may translate into lower stress, anxiety and depression symptoms. Hence, moral resilience may be an important target to preserve mental health in HCWs. Strategies to protect mental health and integrity in HCWs should incorporate interventions which accurately address the root cause of the distress, i.e. the moral dimension, rather than simply treating the symptoms. There is an urgent need to develop and test such interventions and identify whether enhancing moral resilience leads to reduced levels of moral distress and better mental health. The healthcare workforce cannot be manufactured; rather it takes years of training and practice to be competent and gain specialized expertise. Thus, there is an urgent need for healthcare organizations to do more to protect their most valuable asset from the effects of moral distress or risk leaving a void in healthcare delivery by HCWs leaving the profession which may take years to fill.

## Supplementary Information

**Additional file 1.**

**Additional file 2.**

## Data Availability

The datasets used and/or analysed during the current study are available from the corresponding author on reasonable request.

## Abbreviations

HCW: Healthcare Worker
PMDE: Potentially Morally Distressing Event
CHERRIES: Checklist for Reporting Results of Internet E-Surveys
M: Mean
MMD-HP: Measure of Moral Distress for Health Care Professionals
MDS- R: Moral Distress Scale-Revised
RMRS: Rushton Moral Resilience Scale
GAD-7: Generalized Anxiety Disorder Scale
QIDS-SR16: Quick Inventory of Depressive Symptomatology-Self Report
PSS: Perceived Stress Scale
SE: Standard error of B
SD: Standard Deviation
CI: Confidence interval
LL: Lower limit
UL: Upper limit
MEPRA: Mindful, Ethical Practice and Resilience Academy
