# Supplementary material for: The new frontline: exploring the links between moral distress, moral resilience and mental health in healthcare workers during the COVID-19 pandemic
Source: BMC Psychiatry. 2022 Jan 6;22:19. doi: 10.1186/s12888-021-03637-w (PMC8734541; doi:10.1186/s12888-021-03637-w)
Supplement: Supplementary file 1 — Additional file 1. [file 12888_2021_3637_MOESM1_ESM.docx]

**SUPPLEMENTARY MATERIALS**

**Example items of the Rushton Moral Resilience Scale**

In answering the following questions, please consider how you have responded to challenging ethical situations in the last three months in your professional role.

Please rate the following using the following scale:

1=disagree

2= somewhat disagree

3=somewhat agree

4= agree

When a challenging ethical situation can’t be resolved, I find myself “going through the motions” in my job.

I find it challenging to implement the decisions of others when it threatens my values.

I would rather avoid conflict with those who have more authority than I do than act in accordance with my values.

**Other scales descriptions and psychometric properties**

*Cohen’s Perceived Stress Scale (PSS-10)*

The 10 item PSS is used to quantify the perception of stress.(1) Participants answer from a five-point Likert scale, with total scores ranging from 0 – 40, where higher scores indicate greater perceived stress. The Cronbach α and test-retest of the PSS-10 were both reported as greater than 0.70.(2)

*Quick Inventory of Depressive Symptomatology-Self Report, short version (QIDS-SR16)*

The QIDS-SR16 is a questionnaire assessing the nine symptom domains of depression used in the DSM-IV. It contains 16 items for which respondents are asked to rate the severity of symptoms such as sleep disturbances (either reductions or increases in sleep), sadness, appetite and weight changes (either reductions or increases), and restlessness. Scores range from 1-27, with higher scores indicating more severe depression symptoms.(3) The minimum clinically important difference for this questionnaire was found to be ≥ 28.5% (± 28.7%).(4) Based on a meta-analysis, the QIDS-SR16 was found to be unidimensional and to have an internal consistency (Cronbach’s α) ranging from 0.69 to 0.89.(5) This questionnaire was included due to the low mood that may be associated with the imposed isolation.

*Generalized Anxiety Disorder Scale (GAD-7)*

The GAD-7 is a 7 items questionnaire, which is used to screen and assess severity of generalized anxiety disorder. Scores can range from 0 – 21, with a higher score indicating a greater severity. Internal consistency was found to be excellent (Cronbach α = 0.92) and test-retest reliability as good (intraclass correlation = 0.83). The GAD-7 was also found to have good sensitivity (89%) and specificity (82%).(6) The minimal clinically important difference on the GAD-7 was estimated at changes of 4 or greater.(7) This questionnaire was included since several factors related to the pandemic can cause an increase in anxiety (e.g. isolation, the unknown, change).

*Custom-made questions*

Have you ever had a formal diagnosis of any mental disorder (e.g. Anxiety disorder, depression)? Y/N/ I prefer not to respond

If yes: Is it still current? Y / N / Partially

Does your work currently involve:

- - Contact (in person) with the general public?
  - Contact (in person) with people at high risk for COVID-19 {i.e. elderly, chronic illnesses}?
  - Contact (in person) with people who tested positive for COVID-19?

How much do you agree with the following statements

{(NA) (strongly disagree) (neutral) (strongly agree)}

Since the beginning of the outbreak, I have experienced significant levels of support from:

- - my family
  - my employer, colleagues

**REFERENCES**

1. Cohen S, Kamarck T, Mermelstein R. A Global Measure of Perceived Stress. J Health Soc Behav [Internet]. 1983 Dec;24(4):385. Available from: http://www.jstor.org/stable/2136404?origin=crossref

2. Lee EH. Review of the psychometric evidence of the perceived stress scale. Vol. 6, Asian Nursing Research. Elsevier; 2012. p. 121–7.

3. Brown ES, Murray M, Carmody TJ, Kennard BD, Hughes CW, Khan DA, et al. The Quick Inventory of Depressive Symptomatology-Self-report: a psychometric evaluation in patients with asthma and major depressive disorder. Ann Allergy, Asthma Immunol [Internet]. 2008 May;100(5):433–8. Available from: https://linkinghub.elsevier.com/retrieve/pii/S108112061060467X

4. Masson SC, Tejani AM. Minimum clinically important differences identified for commonly used depression rating scales. J Clin Epidemiol [Internet]. 2013 Jul;66(7):805–7. Available from: https://linkinghub.elsevier.com/retrieve/pii/S0895435613000565

5. Reilly TJ, MacGillivray SA, Reid IC, Cameron IM. Psychometric properties of the 16-item Quick Inventory of Depressive Symptomatology: A systematic review and meta-analysis. J Psychiatr Res [Internet]. 2015 Jan;60:132–40. Available from: https://linkinghub.elsevier.com/retrieve/pii/S0022395614002751

6. Spitzer RL, Kroenke K, Williams JBW, Löwe B. A Brief Measure for Assessing Generalized Anxiety Disorder. Arch Intern Med [Internet]. 2006 May 22;166(10):1092. Available from: http://archinte.jamanetwork.com/article.aspx?doi=10.1001/archinte.166.10.1092

7. Toussaint A, Hüsing P, Gumz A, Wingenfeld K, Härter M, Schramm E, et al. Sensitivity to change and minimal clinically important difference of the 7-item Generalized Anxiety Disorder Questionnaire (GAD-7). J Affect Disord [Internet]. 2020 Mar;265:395–401. Available from: https://linkinghub.elsevier.com/retrieve/pii/S0165032719313643

**Figure S1. Correlations between moral resilience and mental health indices.**


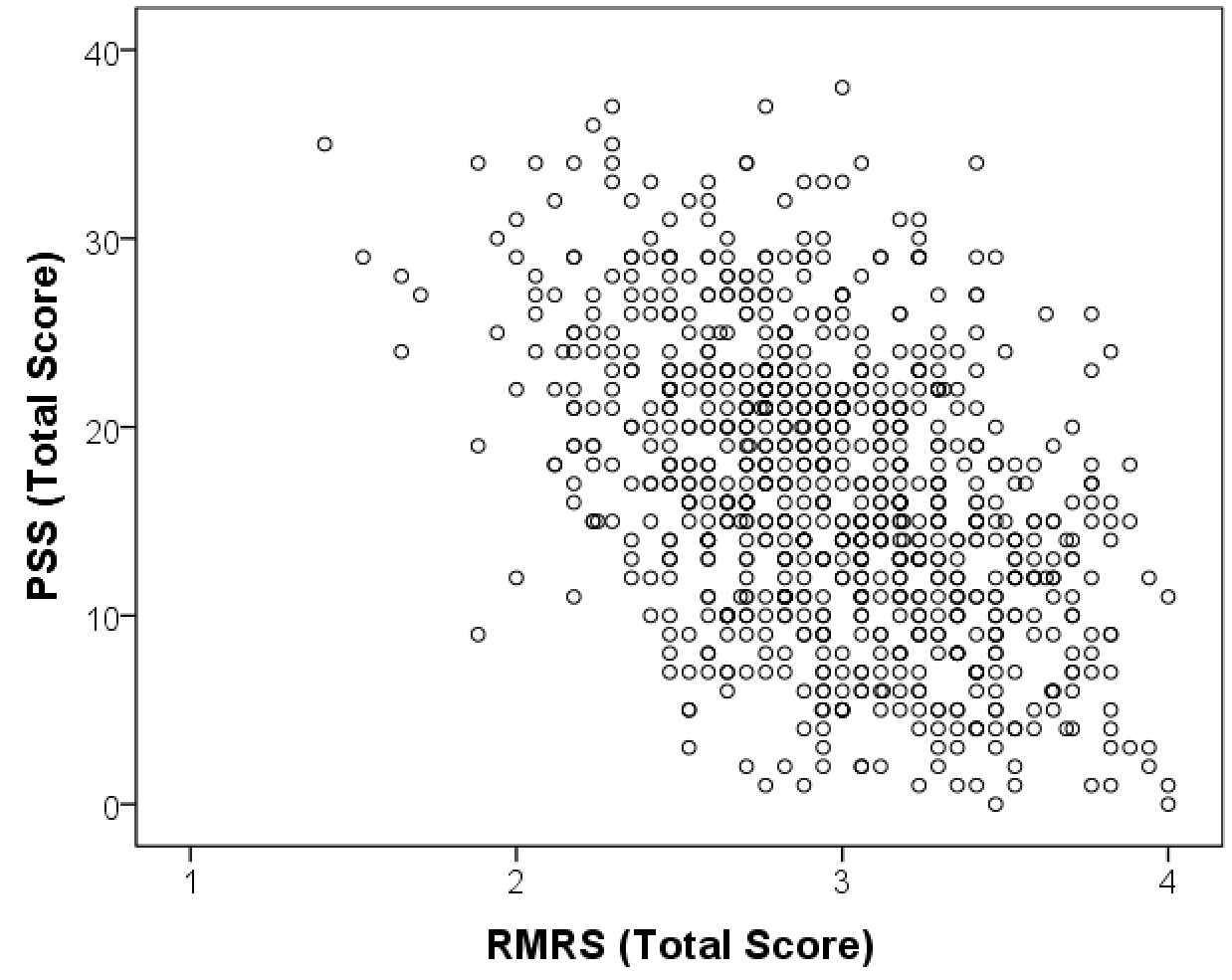


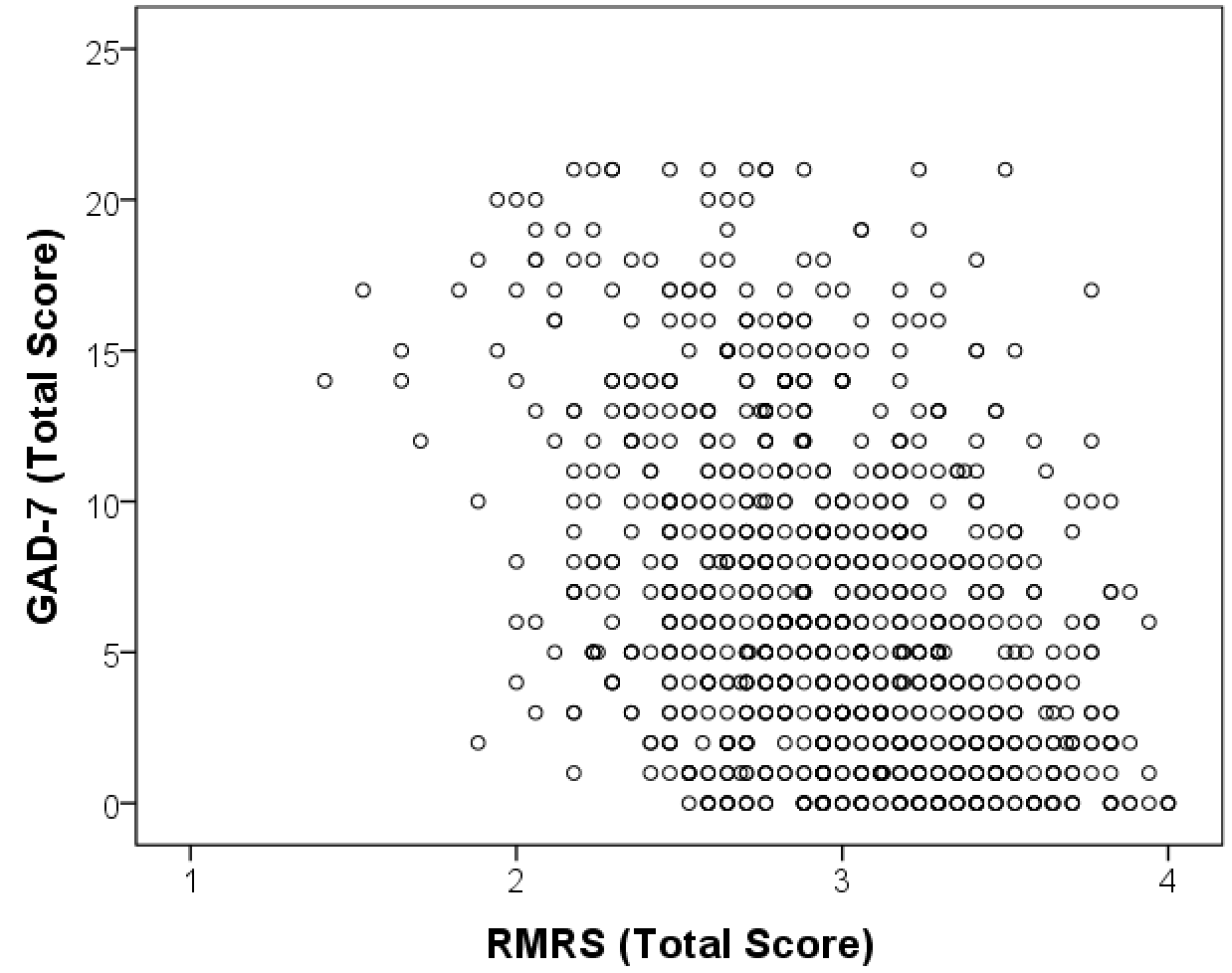


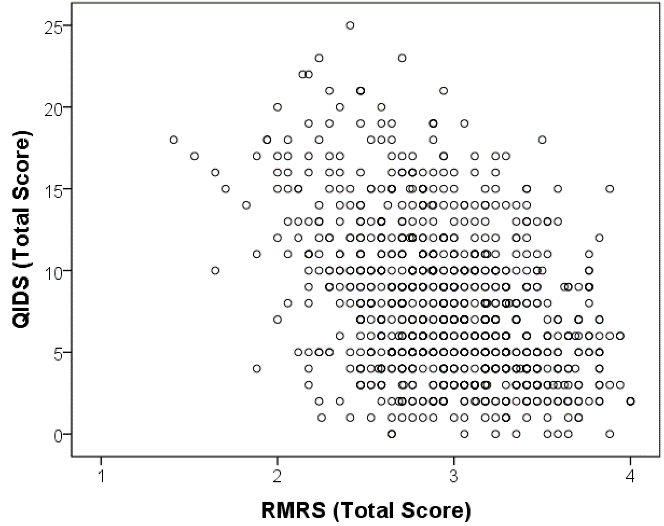


Total PSS score (Perceived Stress Scale, n= 763, *r*=.29, *p*<.001), GAD-7 scores (Generalized Anxiety Disorder Scale, n=829, *r*=.28, *p*<.001), QIDS-SR16 scores (Quick Inventory of Depressive Symptomatology-Self Report, n=801, *r*=.27, *p*<.001) compared with RMRS (Rushton Moral Resilience Scale) total score.
